# Economic benefits of subcutaneous rapid push versus intravenous immunoglobulin infusion therapy in adult patients with primary immune deficiency

**DOI:** 10.1111/j.1365-3148.2012.01201.x

**Published:** 2012-11-20

**Authors:** A Martin, L Lavoie, M Goetghebeur, R Schellenberg

**Affiliations:** 1 St Paul's Hospital Vancouver BC; 2 The LA-SER Group Montreal; 3 Department of Medicine, James Hogg iCapture Centre for Cardiovascular and Pulmonary Research University of British Columbia Vancouver BCCanada

**Keywords:** budget impact model, cost minimisation, IVIG, primary immune deficiencies, SCIG

## Abstract

**Objective:**

The objective of this study is to evaluate the economic benefits of immunoglobulin replacement therapy achieved subcutaneously (subcutaneous immunoglobulin, SCIG) by the rapid push method compared to intravenous infusion therapy (intravenous immunoglobulin, IVIG) in primary immune deficiency (PID) patients from the healthcare system perspective in the context of the adult SCIG home infusion program based at St Paul's Hospital, Vancouver, Canada.

**Materials and methods:**

SCIG and IVIG options were compared in cost-minimisation and budget impact models (BIMs) over 3 years. Sensitivity analyses were performed for both models to evaluate the impact of varying modality of IVIG treatments and proportion of patients switching from IVIG to SCIG.

**Results:**

The cost-minimisation model estimated that SCIG treatment reduced cost to the healthcare system per patient of $5736 over 3 years, principally because of less use of hospital personnel. This figure varied between $5035 and $8739 depending on modality of IVIG therapy. Assuming 50% of patients receiving IVIG switched to SCIG, the BIM estimated cost savings for the first 3 years at $1·308 million or 37% of the personnel and supply budget. These figures varied between $1·148 million and $2·454 million (36 and 42%) with varying modalities of IVIG therapy. If 75% of patients switched to SCIG, the reduced costs reached $1·962 million or 56% of total budget.

**Conclusion:**

This study demonstrated that from the health system perspective, rapid push home-based SCIG was less costly than hospital-based IVIG for immunoglobulin replacement therapy in adult PID patients in the Canadian context.

## Introduction

Primary immune deficiencies (PIDs) are a group of chronic disorders that can affect patients at various ages (Shehata *et al*., 2010). These disorders include agammaglobulinaemia, hyper-IgG syndrome, common variable immunodeficiency (CVID), transient hypogammaglobulinaemia of infancy and selective immunoglobulin deficiencies (Sorensen & Moore, 2000). Prevalence of PID is estimated to be from one in two thousand to one in ten thousand of the general population in the United States (Turvey *et al*., 2009). Insufficient primary antibody production accounts for the majority of PID, which can result in serious opportunistic infections in affected patients (Sorensen & Moore, 2000). Immunoglobulin replacement therapy has become the treatment of choice for PID patients for several decades (Berger, 2008). Immunoglobulin can be administered by intravenous or subcutaneous infusion. Intravenous immunoglobulin (IVIG) infusion is typically performed on a monthly basis in an outpatient setting (hospital), whereas subcutaneous immunoglobulin (SCIG) infusion can be self-administered one or more times a week by the patient at home (Berger, 2004; Lemieux *et al*., 2005). Similar efficacy in preventing infections has been reported between SCIG and IVIG with no difference in severity and length of infections (Chapel *et al*., 2000; Shehata *et al*., 2010). Although these two treatment options are associated with similar efficacy and safety profiles, (Chapel *et al*., 2000) switching from hospital-based IVIG to home-based SCIG was shown to significantly improve health-related quality of life (HRQoL) of adult PID patients (Gardulf *et al*., 2004; Kittner *et al*., 2006; Nicolay *et al*., 2006).

Among the SCIG administration options, a recent US study of a population of PID patients referred to an immunotherapy clinic reported that 71% of patients selected the rapid push method rather than pump infusion administration (Shapiro, 2010). The rapid push method was chosen less often by young children (2–10 years of age) but was the preferred method in teenagers and adults (Misbah *et al*., 2009; Shapiro, 2010).

Healthcare resource utilisation differs markedly between SCIG and IVIG options. European economic studies performed in Sweden (Gardulf *et al*., 1995), Germany (Hogy *et al*., 2005), the UK (Liu *et al*., 2005) and France (Haddad *et al*., 2006; Beaute *et al*., 2010) reported that home-based SCIG was 25–75% less costly for the healthcare system than hospital-based IVIG. A Canadian study reported a cost difference of <10% between the two options (Membe *et al*., 2008). In this study, immunoglobulin product formed 85% of the total cost of IVIG therapy and the same cost was applied to both IVIG and SCIG therapies (Membe *et al*., 2008). In studies from France and UK (Beaute *et al*., 2010; Liu *et al*., 2005), IVIG and SCIG costs were also equivalent but represented a smaller part, 70 and 58%, respectively, of total costs of therapy. In studies from Germany and Sweden, the rationale to include immunoglobulin cost was supported by the lower cost of SCIG compared with IVIG in these countries (Hogy *et al*., 2005; Gardulf *et al*., 1995).

The objective of this study was to explore specifically the economic benefits of the rapid push method for SCIG compared with IVIG from the healthcare system perspective in the context of the adult SCIG home infusion program based at St Paul's Hospital, Vancouver, Canada.

## MATERIALS AND METHODS

### Cost-minimisation analysis

To compare SCIG and IVIG options in the context of the SCIG home infusion program based at St Paul's Hospital in Vancouver, a cost-minimisation analysis was performed, because current clinical knowledge indicates no difference in efficacy or side effect profiles between immunoglobulin therapy given subcutaneously and intravenously (Chapel *et al*., 2000). SCIG delivered as a rapid push rather than as conventional pump infusion was selected for the base case for this study because it is the most commonly used option in the SCIG home infusion program (winged needle butterfly infusion set). This method does not require an infusion pump and delivers SCIG by direct manual push from a syringe over short intervals determined by the patient's comfort level.

**Figure 1:**
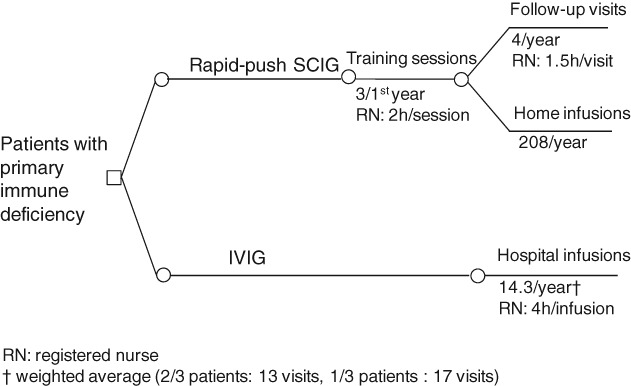
Treatment pathway for the base case models comparing rapid push SCIG and IVIG treatment in primary immune deficiency (PID) based on current practice at the adult SCIG home infusion program, St Paul's Hospital, Vancouver.

The analysis was performed from the perspective of the healthcare system and considered direct medical costs associated with both treatment options over a period of 3 years. The model compared SCIG and IVIG options for administration of immunoglobulin to adult PID patients. Treatment pathway for the base case is depicted in Fig. 1. The SCIG option requires three training sessions and includes four infusions a week using a winged needle butterfly infusion set (208 infusions per year). For IVIG, the base case represents average distribution of two current modalities in use at St Paul's Hospital, i.e. 13 (for two of three of the patients) or 17 visits (for one of three of the patients) annually and a 4-h infusion session each visit.

Resource used for the base-case SCIG and IVIG options for the first 3 years of therapy are considered in the model shown in Table 1 and include supplies for rapid push SCIG and IVIG infusions and personnel. Rapid push SCIG infusion set supplies include butterfly needle, 12-cc syringe, alcohol swabs, gauze and paper tape (6′′). Supplies for IVIG infusion include IV tubing, D5W (5% dextrose in water), Opsite, gauze, IV extension, max plus IV cap, over needle catheter (ONC), saline prefilled syringe and alcohol swabs. SCIG option requires a registered nurse manager (RN/Manager) for training patients (6 h during the first year) and for yearly follow-up (6 h per year). For IVIG, required personnel include a registered nurse (RN), a unit clerk, a scheduling clerk and a ward aid. The nurse for IVIG therapy is on duty for the full infusion period, whereas unit and scheduling clerks and ward aid are paid for 20, 30 and 20 min, respectively, for each visit regardless of duration of the infusion.

**Table 1:** Resource use for base case models

Resources	SCIG	IVIG
First year		
Infusion supplies (day)	208	14·3
RN (h)	—	57·2
RN/manager (h)	12	—
Unit clerk (h)	—	5·72
Scheduling clerk (h)	—	7·15
Ward aid (h)	—	5·72
Second and third years		
Infusion supplies (day)	208	14·3
RN (h)	—	57·2
RN/manager (h)	6	—
Unit clerk (h)	—	5·72
Scheduling clerk (h)	—	7·15
Ward aid (h)	—	5·72

IVIG, intravenous immunoglobulin; RN, registered nurse; SCIG, subcutaneous immunoglobulin.

Unit costs for resources included in the model are shown in Table 2 and were obtained from St Paul's Hospital and the Adult SCIG home infusion program. All costs are in 2011 Canadian dollars. Cost of medication (immunoglobulin) was not included in the model because it is the same for both IVIG and SCIG (Membe *et al*., 2008). As the SCIG winged needle butterfly infusion set does not require a pump, cost of the pump for IVIG was not included as a conservative approach to cost estimation. Parameters considered to have the greatest impact on model output were varied in sensitivity analyses including frequency of visits for IVIG treatment (13–17 visits) and duration of IVIG infusion (4–6 h).

**Table 2:** Unit costs

Parameter	Unit cost (Can$)	Sources
Supplies		
SCIG infusion set Winged needle butterfly (4 times/week)	1·15	Ministry of Health, Product Distribution Centre (2011)
IVIG infusion (total supplies)	13·54	Ministry of Health, Product Distribution Centre (2011)
Personnel (h)		
RN/manager (SCIG treatment)	52·5	British Columbia Nurses' Union (2011)
RN (IVIG treatment)	35·00	British Columbia Nurses' Union (2011)
Unit clerk	19·86	Hospital Employees' Union (2011)
Scheduling clerk	20·77	Hospital Employees' Union (2011)
Ward aid	19·86	Hospital Employees' Union (2011)

IVIG, intravenous immunoglobulin; RN, registered nurse; SCIG, subcutaneous immunoglobulin.

Average wage.

### Budget impact model

A budget impact model (BIM) was developed to evaluate the annual budget impact of using SCIG instead of IVIG in adult PID patients. The epidemiological-based model included BC patients who received IVIG for PID from 1 April 2008 to 31 March 2009 according to BC Central Transfusion Registry (Provincial Health Services Authority, 2011). The analysis was performed from the healthcare system perspective and included the same direct medical costs as those considered in the cost-minimisation analysis over a period of 3 years. It was conservatively assumed that half (50%) of patients on the registry switched from IVIG to SCIG based on data from a US survey, which reported that 75% of IVIG patients were willing to switch to SCIG percent (Ho *et al*., 2008), but that some of those patients were not eligible for SCIG. In the sensitivity analyses, base case results were compared with scenario A: rapid push SCIG versus least expensive option for IVIG (13 visits per year, 4-h infusion per visit); scenario B: rapid push SCIG versus most expensive IVIG option (17 visits per year, 6-h infusion per visit); and scenario C: 75% (instead of 50%) switching to rapid push SCIG versus IVIG option used in the base case (14·3 visits per year, 4-h infusion per visit).

## RESULTS

### Cost-minimisation analysis

Under base case assumptions, SCIG treatment was estimated to cost $1978 per patient over 3 years compared to $7714 per patient for IVIG (Table 3). Using SCIG was less costly than IVIG treatment to the healthcare system by $5736 per patient for the first 3 years of therapy. The main cost difference between the two options was personnel cost, which was greater for IVIG than SCIG: $2378 for IVIG versus $630 for the first year and $315 for the second and third years of SCIG treatment. Yearly cost of disposable infusion supplies was slightly higher in SCIG ($239) compared with IVIG ($194). Results were sensitive to the number of visits during IVIG treatment, with SCIG less costly than IVIG over the first 3 years of therapy (savings of $5035 vs IVIG-13 visits annually and of $7193 vs IVIG-17 visits annually) (Table 4). Results were also sensitive to the duration of IVIG infusion during each visit with $5736 lower cost over 3 years for SCIG versus IVIG (base case 4-h infusion) and $8739 lower cost when a 6-h infusion was employed.

**Table 3:** Cost-minimisation model results (base case)

Costs per patient	SCIG (Can$)	IVIG (Can$)	Increment: SCIG − IVIG (Can$)
First year			
Infusion supplies	239	194	46
RN or RN/manager salary	630	2002	−1372
Other personnel salary			
Unit clerk	—	114	
Scheduling clerk	—	149	
Ward aid		114	
Total personnel salary	630	2378	−1748
Total (first year)	869	2571	−1702
Second year		
Infusion supplies	239	194	46
RN or RN/manager salary	315	2002	−1687
Other personnel salary			
Unit clerk	—	114	
Scheduling clerk	—	149	
Ward aid	—	114	
Total personnel salary	315	2378	−2063
Total (second year)	554	2571	−2017
Third year		
Infusion supplies	239	194	46
RN or RN/manager salary	315	2002	−1687
Other personnel salary			
Unit clerk	—	114	
Scheduling clerk	—	149	
Ward aid	—	114	
Total personnel salary	315	2378	−2063
Total (third year)	554	2571	−2017
Total cost per patient (3 years)	1978	7714	−5736

IVIG, intravenous immunoglobulin; RN, registered nurse; SCIG, subcutaneous immunoglobulin.

**Table 4:** Sensitivity analyses for the cost-minimisation model

Parameter	Range	Increment SCIG–IVIG(Can$ range)
Number of annual visits for IVIG	13–17	−5035 to −7193
Time of IVIG infusion per visit (h)	4–6	−5736 to −8739

IVIG, intravenous immunoglobulin; SCIG, subcutaneous immunoglobulin.

### Budget impact model

The population of PID patients on the BC registry for the fiscal year 2008–2009 was 456 (Provincial Health Services Authority, 2011). Under a conservative estimate of 50% of patients switching from IVIG of PID to SCIG, budget impact for the first 3 years after implementation of SCIG therapy was estimated to be $1·308 million less for the base case scenario (Table 5). This represents a cost reduction of 37% of the total budget (all patients under IVIG therapy). This reduction in cost was $1·148 million when SCIG was compared to the least expensive IVIG option (Scenario A: 13 visits and 4-h infusion) and $2·454 million when it was compared to the most costly IVIG option (Scenario B: 17 visits and 6 h infusion). This represents 36 and 42% cost reduction, respectively, of the total budget. If the proportion of patients switching to SCIG was increased to 75% rather than 50% (Scenario C), $1·962 million less would be required compared to the IVIG base case scenario (14·3 visits and 4-h infusion), a cost reduction of 56% of total budget.

**Table 5:** Budget impact model results (base case and sensitivity analyses) (Can$)

Base case	Baseline	Year 1	Year 2	Year 3	Total 3 years
% Switching (IVIG to SCIG)	—	50	50	50	—
Number of patients IVIG	456	228	228	228	—
Number of patients SCIG	0	228	228	228	—
Total cost IVIG ($)	1 172 525	586 262	586 262	586 262	**1 758 787**
Total cost SCIG ($)	0	198 178	126 358	126 358	**450 893**
Incremental cost ($)	—	−388 085	−459 905	−459 905	−**1 307 894**
% Increment	—	−33	−39	−39	−37
Sensitivity analysis A (IVIG 13 visits—4 h)					
Total cost IVIG ($)	1 065 931	532 966	532 966	532 966	1 598 897
Total cost SCIG ($)	0	198 178	126 358	126 358	450 893
Incremental cost ($)	—	−334 788	−406 608	−406 608	−1 148 004
% Increment	—	−31	−38	−38	−36
Sensitivity analysis B (IVIG 17 visits—6 h)					
Total cost IVIG ($)	1 936 550	968 275	968 275	968 275	2 904 826
Total cost SCIG ($)	0	198 178	126 358	126 358	450 893
Incremental cost ($)	—	−770 098	−841 918	−841 918	−2 453 933
% Increment	—	−40	−43	−43	−42
Sensitivity analysis C (switching 75%)					
Number of patients IVIG	456	114	114	114	—
Number of patients SCIG	0	342	342	342	—
Total cost IVIG ($)	1 172 525	293 131	293 131	293 131	879 393
Total cost SCIG ($)	0	297 266	189 536	189 536	676 339
Incremental cost (4)	—	−582 127	−689 857	−689 857	−1 961 841
% Increment	—	−50	−59	−59	−56

IVIG, intravenous immunoglobulin; SCIG, subcutaneous immunoglobulin.

## DISCUSSION

This study provides an economic evaluation of rapid push SCIG compared with IVIG treatment in adult PID patients from the perspective of the healthcare system. A cost-minimisation analysis demonstrated that rapid push SCIG is less costly than IVIG ($1978 compared to $7714 per patient for the first 3 years of treatment), primarily because of less hospital personnel costs. This represents a cost difference between SCIG and IVIG treatments over 3 years of $5736 or a 74% reduction. In contrast with a previous Canadian economic study (Membe *et al*., 2008) the cost of immunoglobulin, which is the same for SCIG and IVIG in Canada, was not taken into account in the current analysis. Our study provides a comparison between the rapid push SCIG and IVIG options focused specifically on supplies and human resources, reflecting the context of the adult SCIG home infusion program based at St Paul's Hospital in Vancouver.

Results of this study are in agreement with one from the UK in which immunoglobulin costs for IVIG and SCIG were equivalent, which reported that SCIG was less costly than IVIG because hospital costs during IVIG therapy were greater than infusion pump and material required for SCIG treatment (Liu *et al*., 2005). In the UK study, annual costs were reduced by 88% for home-based SCIG compared with hospital-based IVIG with infusions every 3 weeks (Liu *et al*., 2005). In our study, 3-year costs of SCIG therapy compared with IVIG treatment with the same frequency (every 3 weeks) were reduced by 78%. Other European studies comparing home-based SCIG and hospital-based IVIG reported that the SCIG option was less costly principally because of less expensive immunoglobulin for SCIG (55–82%) (Gardulf *et al*., 1995; Hogy *et al*., 2005) or to 31% less immunoglobulin required to achieve therapeutic serum levels in SCIG compared with IVIG (Beaute *et al*., 2010).

Our study also evaluated the budgetary impact of switching 50% of the 456 adult PID patients in the BC Transfusion Registry from IVIG to SCIG treatment and found a reduction of $1·3 million over 3 years for the healthcare system in BC. A pan-Canadian budget impact analysis, which considered the cost of immunoglobulin, reported annual savings for the healthcare systems of $6 million ($5·6 million 2007Can$ converted to 2011Can$) across Canada if 75% of 5460 adult patients switched from IVIG to SCIG treatment (Ho *et al*., 2008). In a German budget impact analysis, annual savings of $43 million ($28 million 2003€ converted to 2011Can$) from the perspective of the German statutory health insurance were reported for switching 60% of 2940 adult patients receiving IVIG treatment to SCIG but in this case immunoglobulin was much less costly for SCIG compared with IVIG (Hogy *et al*., 2005). The BIM presented here was sensitive to the proportion of patients switching from IVIG to SCIG suggesting the most economically efficient use of budget might be achieved by greater utilisation of SCIG therapy when clinically appropriate. Although difficult to quantify, freeing up of resources resulting from SCIG program could allow handling a greater number of interventions requiring IV infusions, such as chemotherapy.

From a patient perspective, the autonomy associated with SCIG administration translates into improved quality of life for PID patients. Studies using the Medical Outcomes Study 36-Item Short Form Health Survey (SF-36) questionnaire to assess HRQoL and the Life Quality Index (LQI) to evaluate treatment satisfaction (TS) have reported that switching from hospital-based IVIG to home-based SCIG improved both HRQoL and TS for adult PID patients significantly (Gardulf *et al*., 2004; Nicolay *et al*., 2006). In addition, SCIG delivered by rapid push has recently been shown to be preferred by adult PID patients who were given the choice (Misbah *et al*., 2009; Shapiro, 2010).

Typically, patients used SCIG delivered by the rapid push method 3·11 times a week for a 5–20 min infusion at one site each time achieving similar serum immunoglobulin levels and safety profile compared to patients who has chosen conventional pump infusion method (Shapiro, 2010). In this study, rapid push SCIG was delivered four times a week with similar infusion times.

These results should be considered in light of study limitations. Because the most commonly used SCIG options in the SCIG home infusion program at St Paul's hospital does not required a pump, the cost of the SCIG pump was not included in the analyses. Considering a pump-based SCIG option would still result in savings to the healthcare system, estimated at $1621 per patient and $369 665 in the budget impact analysis for the first 3 years of therapy compared to the IVIG treatment used in the base case even with the conservative approach of no cost for IVIG pump. This estimate assumed a weekly cost of infusion supplies of $17·90 (Quadfurcated tubing safety Sub-Q infusion set) (RMS Medical Products, Chester, NY, US); that a SCIG pump lasts for 5 years and that two pumps are recommended for each patient (Hogy *et al*., 2005; Liu *et al*., 2005); that the type of infusion pump currently used at St Paul's Hospital costs $450·00 (Freedom 60 Syringe driver), and adapting tubing for the pump costs $500·08 per year [F900 Precision Infusion Tubing set (box of 50)] (RMS Medical Products, Chester, NY, US).

Another limitation relates to the fact that the models were designed to explore economic impact from the healthcare system perspective and therefore did not take into account costs borne by patients (parking and travel) and indirect costs such as productivity loss from patient and caregiver's time devoted to treatment. However, given the flexibility of home-based SCIG administration when considering the time, frequency and speed of infusion, patients' borne costs are minimal compared to hospital-based IVIG therapy. Finally, we did not consider the population of patients with secondary immune deficiencies which result from a variety of factors including infectious agents, drugs, metabolic diseases and environmental conditions (Chinen & Shearer, 2010).

In conclusion, this study estimated that replacing IVIG with rapid push SCIG in 50% of adult PID patients resulted in 3-year savings for the healthcare system of $5736 per patient, representing reduced costs of $1·3 million for the population of adult PID patients in British Columbia. Although focusing on the adult SCIG home infusion program based at St Paul's Hospital in Vancouver this study is in line with the current Canadian clinical practice and it can be expected that results would be generalisable to other Canadian settings. Hence, in addition to increased patient autonomy provided by SCIG compared with IVIG, shorter infusions better adapted to daily life of patients, and no need for a pump device, rapid push SCIG not only provides an improved option for patients but also results in significantly reduced costs from the healthcare system perspective in the Canadian context for immunoglobulin replacement therapy in adult PID patients. Further research incorporating societal costs and the population of secondary immune deficiencies patients could help achieve better insight into the economic consequences to be derived from use of the rapid push method for patients with immunoglobulin deficiency.
